# On the arts and humanities in medical education

**DOI:** 10.1186/s13010-021-00102-0

**Published:** 2021-06-30

**Authors:** Danielle G. Rabinowitz

**Affiliations:** 1 Department of Pediatrics, Boston Children’s Hospital, 300 Longwood Avenue, Boston, MA 02115 USA; 2 Department of Pediatrics, Boston Medical Center, Boston, MA USA; 3 Harvard Medical School, Boston, MA USA

**Keywords:** Medical humanities, Medical humanism, Medical education, Medicine and arts

## Abstract

This paper aims to position the birth of the Medical Humanities movement in a greater historical context of twentieth century American medical education and to paint a picture of the current landscape of the Medical Humanities in medical training. It first sheds light on the model of medical education put forth by Abraham Flexner through the publishing of the 1910 Flexner Report, which set the stage for defining physicians as experimentalists and rooting the profession in research institutions. While this paved the way for medical advancements, it came at the cost of producing a patriarchal approach to medical practice. By the late 1960s, the public persona of the profession was thus devoid of humanism. This catalyzed the birth of the Medical Humanities movement that helped lay the framework for what has perpetuated as the ongoing incorporation of humanistic subjects into medical training. As we enter a time in medicine in which rates of burnout are ever-increasing and there are growing concerns about a concomitant reduction in empathy among trainees, the need for instilling humanism remains important. We must consequently continue to consider how to ensure the place of the Medical Humanities in medical education moving forward.

In his address to the greater medical community in an article published in August, 2017, president and chief executive officer of the Arnold P. Gold Foundation, Dr. Richard Levin, speaks of the challenges inherent in becoming a practicing physician today. In particular, he notes the growing degree of physician burnout, lack of satisfaction in the workplace, and depression among those in the medical field. In conjunction with this, he shares the shocking statistic that the rate of physician suicide is over two times the national average. In Levin’s view, which mirrors that of other advocates of the arts and humanities in medicine, the remedy for what has become the current state of affairs in medical practice rests in ensuring the deliberate infusion of humanism into medical training. He contends that “only when we care for the clinicians themselves—when the whole health team is healthy ourselves—can we care for the patient optimally” [1]. Levin’s objectives echo what has become a decades-long dialogue among those in medicine as to the best approach to utilizing Medical Humanities curricula to help shape doctors who are both inherently happy and empathic. This paper will therefore aim to position the birth of the Medical Humanities movement in a greater historical context of twentieth century American medical education and to paint a picture of the current landscape of the Medical Humanities in medical education. In doing so, the hope will be to help the reader better understand the need for ongoing reform and to stimulate further discussion as to the way forward with regard to developing the best means through which to ensure the place of the Humanities in medicine.

The framework for the current model of American medical education in the United States was laid through the publishing of the Flexner Report in 1910. The document represented a milestone in a lengthy project conducted by Abraham Flexner and supported by the American Medical Association in conjunction with the Carnegie and Rockefeller Foundations that aimed at “sweeping clean the medical system of substandard medical schools that were flooding the nation with poorly trained physicians” [2]. It called for the closure of over a hundred medical institutions based on subpar facilities and course offerings, urged standardization of prerequisite courses for medical education, necessitated faculty engagement in scientific research, and set the stage for improved regulation of state-based medical licensure [3].

In developing his agenda, Flexner sought to emulate the German system of medical education that had seen reform in the latter part of the nineteenth century and that had given rise to research laboratories and the notion of the physician as experimentalist. He therefore sought to instill much more formally into the American system the “use [of] inquiry and research to advance the practice of medicine” [4]. Yet, even at the time of implementation, the Flexnerian viewpoint saw opposition. One of the most vocal voices in this debate was Sir Dr. William Osler (1849–1919), who feared that the dogmatic standardization of medical education and the strict tying of the physician to the research laboratory would be detrimental to the doctor-patient relationship and to the humanistic elements of medical practice. He was particularly afraid that “teacher and student [would] chase each other down the fascinating road of research, forgetful of those wider interests to which a hospital must minister” [2]. Drawing on this, in his 1919 address to the Classical Association on The Old Humanities and the New Science just prior to his death, he woefully lamented the “unhappy divorce” between Science and the Humanities, which had caused “young men … applying themselves early to research … [to] get into backwaters far from the mainstream” [5].

Despite Osler’s pleas to ensure the continued unification of medical practice with humanism, the Flexner report served as the dominant influence in the shaping of modern American medical education. Training of physicians therefore became firmly rooted in research institutions, paving the way for the burgeoning of medical advancements. In the early part of the twentieth century, physicians practicing in the United States experienced what numerous historians have coined “the golden age of medicine,” adopting a new “paradigm … of cause and cure … with its strong ties to laboratory science and technological apparatus” [6].

The elevation in power of the medical profession, however, was not without negative implications. First and foremost, it enabled the development of a patriarchal approach to medical practice. The physician’s “arrogance and abuses of individual rights in both the clinic and in human experimentation” marred the relationship between doctor and patient [6]. Physician paternalism ran rampant, and the doctor became regarded by the public as largely “robot [ic]” and as “automatic … forget [ting] that the people they treat are human beings” [7]. This ultimately gave rise, by the late 1960s, to the development of patient rights advocacy, representing a response to ethical missteps among doctors, both in the United States and abroad, in the decades prior [8]. In particular, this came on the heels of over twenty years of growing mistrust of doctors as a whole –beginning with the exposure of the involvement of German physicians in egregious and horrific human experimentation during World War II through the worldwide broadcasting of the Doctor’s Trials held in Nuremberg, Germany between 1946 and 1947 [9], and coming to a fore in 1972 following the public spotlight on the inhumane practices of physicians at the Tuskegee Institute, which had studied from as early as 1932 the ramifications of untreated syphilis on a poor, black population in Mississippi despite the widespread acknowledgement of the curative effects of Penicillin by as early as the 1940s [10].

In light of this, the American medical community, too, began to make strides toward recrafting their public image. In 1966, Dr. Henry Knowles Beecher (1904–1976) published his seminal work in the New England Journal of Medicine on “Ethics and Clinical Research” in which he urged those involved in medical research at large to adopt a significantly more stringent ethical standard for scientific experimentation, shedding light on what he deemed to be “troubling practices” nationwide [11]. Discussions centered on reinfusing humanism into the medical profession also began during this time period. At the helm of what came to be known as the Medical Humanities movement was Dr. Edmund Pellegrino (1920–2013), an American bioethicist who recognized the importance of re-instilling into the medical world what Osler had feared all too well would be lost with the implementation of the Flexner report at the turn of the twentieth century.

Pellegrino served as the first chairman of the Society of Health and Human Values’ Institute on Human Values in Medicine, which was proposed in 1969 following the publishing by Dr. Lorraine Hunt of “A Survey of the Current Status of Humanities Program in Selected Medical Schools,” a study supported jointly by the Association of American Medical Colleges [12] and the National Endowment for the Humanities. The Institute signified a national response to “those in medicine … concerned with issues involving human values,” and served as an invited forum in which medical scholars could work toward the development of a more formal framework for integration of Humanities into medical education [13]. In his opening remarks at the Institute’s first official meeting in 1971, Pellegrino spoke of the role of “medical progress … in open [ing] up difficult questions about the relationships of medicine and technology to human values—matters of the utmost concern to the humanist [13]. In doing so, he urged those present to consider the importance of “the application of the humanistic disciplines—like literature, history, philosophy—to the matter of medicine. To the way we practice medicine, to the way we behave as physicians, to our writing, to our whole attitude on the profession itself” [14]. Between 1971 and 1981, the Institute produced 17 reports offering ideas on how to appropriately introduce Humanities coursework into medical education [15].

In response to the profound efforts of Pellegrino and his colleagues, medical schools across the nation began to more ubiquitously incorporate humanities courses into their curricula. Yet, the Institute lay the groundwork for what became a patchwork of implementation strategies. This has created a situation in which there is, even into the present day, an ongoing debate as to the most beneficial approach to teaching the Humanities to medical students—and consequently no pervasive single approach that has been adopted across medical schools. Relatedly, it is important to note that the first definitive textbook in the Medical Humanities, which provides one possible framework through which to examine medical history, philosophy, and the like, was only published in 2014 [16]. In a 2017 article on *Integrating Humanities Curricula in Medical Education: A Needs Assessment*, authors Taylor, Lehmann, and Chisolm, speak to the lack of clarity surrounding the effectiveness of various Humanities-based courses despite the widespread recognition of the fact that exposure of medical students to this coursework “encourage [s] reflective practice and promote [s] the practice of holistic patient care” [17]. After searching the existing literature on medical humanities, they ultimately found that 69% of articles were reflective papers and 31% were those highlighting novel educational interventions. Of the intervention-based articles, however, only 10 out of the 48 provided data on impact utilizing either a qualitative or quantitative approach. In addition, most of the included courses were based around literature or ethics, with many fewer exploring the role and impact of visual and/or performing arts. The paper consequently concludes that their review “identified a number of significant gaps in the literature, the most important being a lack of outcome data,” speaking to inability of educators in various medical schools to effectively select the most high-value coursework for use in medical education contexts [17]. Relatedly, in their paper on *Evaluating the Impact of the Humanities in Medical Education*, Authors Schwartz et al. similarly note that despite “many humanities electives and courses [being] offered in … medical school curricula, measuring and quantifying their impact has proven challenging because the courses are diverse in content and goals” [18]. They therefore urge medical educators to develop better strategies that capture impact of various programs, with a particular emphasis on more thoughtful consideration of the outcomes that seem most desirable and sought after with respect to the coursework itself [18].

The lack of standardization in approach to Humanities-based curricula in medical schools has become central to a broader discussion regarding the importance of maintaining said curricula in medical education as launched by the AAMC in conjunction with the National Endowment for the Humanities, the same two organizations that united over 40 years ago to address similar issues under Pellegrino’s leadership. In July, 2017, a conference was held to strategically “build a case to medical educators that [studying the arts and Humanities] is integral to what we do [as physicians]” [19]. This came on the heels of results identified through an AAMC-lead curriculum inventory that highlighted that in the 2015–2016 academic year, only 119 American medical schools had a mandatory Medical Humanities course versus 103 offering electives (AAMC Curriculum Report on Medical Humanities). This underscored the lack of a “deep, sustained, foundational, across-the-board incorporation into all [American] medical schools” [19].

In his 2014 article on *A Complete Medical Education Includes the Arts and Humanities*, Dr. David Jones writes of the ongoing challenges of cementing curricular reform with regard to Medical Humanities. He highlights the continued difficulty of medical professionals in answering the question as to how exactly to effectively infuse into the doctor-to-be “empathy … compassion, sincerity, dedication, [and] professionalism,” traits that many staunch supporters of required arts and humanities coursework have argued can be gleaned from the medical student’s involvement in such activities [20]. At the same time, however, he juxtaposes this concern with the widespread acceptance and acknowledgement of the powerful role of the Medical Humanities, as echoed in a recent report by the American Academy of Arts and Sciences, in “foster [ing] creativity, appreciation of our commonalities and our differences, and knowledge of all kinds,” which are all imperative attributes of a person in the field of caregiving [20]. The balancing of these two viewpoints is further complicated, however, by the shift within the frame of the AAMC toward the use of “competencies, milestones, and empirical assessment of educational outcomes” to ensure some degree of calibration of medical education across schools [20]. As noted above, when it comes to defining the contribution of Medical Humanities to the achievement of these competencies, the lack of ease in ascertaining outcome measures makes this particularly challenging. There are existing novel models that measure impact in the realm of the arts, most notably that of the coursework offered through a collaboration between Brigham and Women’s Hospital and the Museum of Fine Arts, Boston, in which the observation skills of resident physicians in the exam room were shown to markedly improve pre- and post-exposure to a program on the study of Renaissance portraits, but there is a paucity of literature overall on such interventions [21].

We are in a moment in medicine in which the demands on physicians are ever-increasing, as new technologies and scientific discoveries are rapidly changing the medical landscape. Rates of burnout are skyrocketing, as are studies showing that empathy among medical students is becoming progressively stunted [22]. In the time that we are now in, it is interesting to consider more critically Flexner’s perspectives on the arts and humanities given his individual role in shaping what has since grown into the rigorously scientific underpinnings of medicine today. In an article that he published on the importance, in his view, of the pursuit of “Useless Knowledge” in Harpers Magazine in 1939, Flexner states:

I have spoken of experimental science … but what I say is equally true of music and art and of every other expression of the untrammeled human spirit. The mere fact that they bring satisfaction to an individual soul bent upon its own purification and elevation is all the justification that they need [23].

This in many ways reflects the notion of ““*L’art pour l’art*“ (art for art’s sake) as promulgated by 19^th^ Century French author Theophile Gautier in response to societal pressures placed on artists that dictated development of moral justifications for their art. Perhaps, therefore, Flexner’s personal views on the importance of the Humanities ought to reframe our consideration of the driving rationale behind the importance of continued integration of Medical Humanities into medical education. Is the focus on and dogged pursuit of the development of outcome measures and parameters for effectiveness necessary altogether in the context of this coursework? Or do we already have argument enough in the recognition of the “satisfaction … and elevation” that the arts and humanities bring to physicians, serving as sufficient justification for use in facilitating the crafting of a new generation of empathic, holistically-oriented providers.

## Data Availability

All cited materials are available for viewing in print and/or online.
